# The Impact of Threat Appeals on Fear Arousal and Driver Behavior: A Meta-Analysis of Experimental Research 1990–2011

**DOI:** 10.1371/journal.pone.0062821

**Published:** 2013-05-17

**Authors:** Rachel N. Carey, Daragh T. McDermott, Kiran M. Sarma

**Affiliations:** 1 School of Psychology, National University of Ireland, Galway, Galway, Ireland; 2 Department of Psychology, Anglia Ruskin University, Cambridge, United Kingdom; University of Minnesota, United States of America

## Abstract

The existing empirical research exploring the impact of threat appeals on driver behavior has reported inconsistent findings. In an effort to provide an up-to-date synthesis of the experimental findings, meta-analytic techniques were employed to examine the impact of threat-based messages on fear arousal and on lab-based indices of driving behavior. Experimental studies (*k* = 13, *N* = 3044), conducted between 1990 and 2011, were included in the analyses. The aims of the current analysis were (a) to examine whether or not the experimental manipulations had a significant impact on evoked fear, (b) to examine the impact of threat appeals on three distinct indices of driving, and (c) to identify moderators and mediators of the relationship between fear and driving outcomes. Large effects emerged for the level of fear evoked, with experimental groups reporting increased fear arousal in comparison to control groups (*r* = .64, *n* = 619, *p*<.01). The effect of threat appeals on driving outcomes, however, was not significant (*r* = .03, *p* = .17). This analysis of the experimental literature indicates that threat appeals can lead to increased fear arousal, but do not appear to have the desired impact on driving behavior. We discuss these findings in the context of threat-based road safety campaigns and future directions for experimental research in this area.

## Introduction

Risky and reckless driving behavior is a central concern for law enforcement and road safety agencies world-wide, and is linked to increased road traffic collisions (RTCs), injuries and fatalities. The World Health Organisation has estimated that, by 2020, RTCs will be the third largest cause of death worldwide. It is unsurprising, then, that road safety organizations have gone to considerable effort and expense to wage mass-media campaigns aimed at changing driver practice. One approach that is often employed in these campaigns is the use of threat-based advertising, or ‘threat appeals’.

Threat appeals have been widely utilized in road safety advertising campaigns in an attempt to discourage risky driving, and typically present graphic representations of the death and injury that may occur as a result of a RTC. Despite their prevalence, threat appeals have provoked controversy for both ethical and practical reasons, and their effectiveness as a form of persuasive communication has been questioned [1]. This is partly due to inconsistent findings in the empirical research. Specifically, while some papers argue that threat appeals can be highly effective, provided a number of conditions are met [e.g. 2], findings from other studies suggest that they can lead to maladaptive responses, and may even provoke an increase in the risky behavior [e.g. 3], [4].

These inconsistencies in the literature have made it difficult to authoritatively advise road safety practitioners as to ‘what works’ when designing threat appeals, as well as whether or not such appeals should feature prominently in road safety communications. Meta-analyses of the research investigating the impact of threat appeals on driver behavior can help clarify the utility of this approach. The central objective of this paper is to present the first meta-analysis of the experimental research that has directly tested the impact of threat appeals on indices of driving.

### Existing evidence

There are two important points about the existing evidence-base that suggest a meta-analysis of this nature is warranted. First, while there have been a number of high-quality meta-analyses published relevant to driver behavior, these analyses are of limited value in informing our understanding of road safety threat appeals. This is because no meta-analysis has specifically examined the impact of threat appeals on driving. Instead, these studies have tended to combine multiple risky behaviors (e.g. non-condom use and smoking [5]) in their analyses, or have combined different types of road safety campaigns (e.g. threat-based and education-based [6]) to ascertain if mass-media campaigns, in general, are effective. Thus, the results of this body of empirical literature may lack predictive validity in informing our predictions as to the impact of threat appeals on driving behavior. Second, the experimental literature in the area has reported inconsistent findings, reflecting variations in the robustness of the designs deployed, the moderators included, and the dependent variables used as indices of driving behavior.

Turing first to the meta-analyses, some important findings in previous analyses have shaped the way we think about threat appeals. For instance, a number of meta-analyses and systematic reviews have examined the impact of road safety advertising in general (i.e. not threat-specific) on driving outcomes. One meta-analysis suggested that mass-media campaigns significantly reduce drink-driving [7], and a recent paper estimated that, allowing for heterogeneity, road safety campaigns coincided with a 9% reduction in RTCs [6]. This would suggest that mass media approaches can work in reducing negative outcomes, although it does not allow for specific inferences to be made about threat appeals.

Several meta-analyses [e.g. 8], [9] have analyzed the threat appeal literature, examining the effect of this type of message on health-relevant outcomes in general (i.e. combining multiple behaviors, such as smoking, risky sexual behavior, breast self-examination, sunscreen usage and drug taking, within individual meta-analyses). A meta-analysis of threat appeals, conducted by Witte and Allen [2], demonstrated that high-threat messages with high response efficacy (i.e. the recommended behavior in the message is likely to be seen as effective) and high self-efficacy (i.e. the recommended behavior is likely to be seen as achievable by the individual) produce the greatest behavior change.

A recent meta-analysis by Peters and colleagues [5] provided further support for the role of perceived efficacy (made up of response efficacy and self-efficacy) in moderating the effectiveness of threat appeals. The authors highlighted the importance of self-esteem, suggesting that the risky behavior of those who derive self-esteem from that behavior may be exacerbated following exposure to a threat appeal, a finding echoed in several recent experimental papers [3], [4], [10]. The authors suggest that persuasive communications that focus on enhancing perceived efficacy, or other relevant variables, are more likely to create positive behavior change than those that do not focus on these variables.

De Hoog and colleagues have proposed a stage model of threat appeal-processing, one facet of which proposes that threat appeals can have different effects on attitudes and behavior [9]. Positive attitudes towards a preventative act, they propose, are based on an objective analysis of the risks posed by the threat, and thus influenced by factors such as argument quality. Behavior, or intended behavior, on the other hand, requires that we feel vulnerable to the negative consequences of non-action. Their meta-analysis of 105 studies found support for this position, with factors linked to controlled cognitive processing (argument quality and severity of consequences of non-action) linked to positive attitudes towards the recommended actions. Perception of vulnerability was not linked to attitudes, but was an important determinant of behavior intention and behavior change.

Overall, meta-analytic research in this area has suggested that threat appeal messages can be effective, provided that they are evidence-based. There is, however, an important limitation to this research when attempting to apply it to road-safety threat appeals. Previous analyses have either examined threat appeals, used to promote a broad range of health behaviors, and are thus not specific to driving, or they have investigated a broad range of road safety messages, and are not specific to threat appeals. Consequently, it is unclear to what extent the findings from these studies can be used in informing our understanding of the utility of road-safety threat appeals.

As noted earlier, the experimental literature offers a useful source of evidence on the causal relationship between exposure to threat appeals and driving outcomes, but has reported inconsistent findings. Researchers have offered a number of reasons for this [11]. One explanation is that experimental studies have operationalized and measured independent and dependent variables in different ways [5]. In a laboratory setting, driving tends to be measured through self-report ‘intention to act’ measures [e.g. 4], driving simulators [e.g. 12] or simulated driving scenarios, presented through digital video images (e.g. Video Speed Test [VST]; [ 13], [14], [15], [16,17]). The ecological validity of such dependent variables has been criticized [e.g. 18], [19], as has the potential for social desirability biases to confound results [20]. The concern here is that discordant findings in the experimental literature may be due, at least in part, to the deployment of driving measures that differ in terms of their sensitivity to the effects of the experimental manipulations. In a meta-analysis, this could be evident as a cluster of significant findings, or comparable strong effects, among studies that used one specific type of dependent variable.

### Influential theory

Another possible explanation for the contradictory findings in the literature is that our conceptualization of the causal relationship between the fear emotion and behavior has been either erroneous, or overly simplistic. Baumeister and colleagues [21], [22] have argued that, while psychology has long held that emotions directly cause behavior (i.e. a direct causation model), in reality the evidence-base for this position is ‘neither extensive nor convincing’ [21, p. 171]. Other researchers have reached the same conclusion. Schwarz and Clore [23] asserted that the direct effects of emotion are ‘more mental than behavioral’, and that the onset of fear does not, in itself, predict ‘whether people will sell their stocks, listen to the weather report, or start running’ (p. 402).

Baumeister and colleagues [21] suggest that the link between fear and behavior is complex. They propose that a key function of emotion is to provide feedback as to the appropriateness of different actions. For example, an individual may experience fear after engaging in a dangerous driving maneuver. This negative emotional state, and the desire to avoid a similar state in the future, forces the individual to reflect on the initial decision to accelerate past the vehicle, and to identify lessons (if-then rules) to avoid a repeat of the action (i.e. dangerous-bend approaching = do not pass). These rules, according to Baumeister, are stored with an ‘affective residue associating’ (p. 173) guilt with that action, thereby guiding future behavior. Their point is that the main proximal impact of emotions, such as fear, is on cognitions, and not on behavior.

While it is difficult to directly apply this kind of feedback model to our understanding of threat-appeals, it raises the possibility that the impact of fear, elicited through such appeals, on behavior is mediated or moderated by the extent to which the fear results in these cognitions. It is cognitions, then, including those involving lesson-learning and if-then rules, that can impact on later decisions.

Another theoretical approach which also highlights the role of cognitions is the Extended Parallel Process Model [EPPM; 24]. The EPPM, an extension of Leventhal's [25] Parallel Process Model, was developed specifically as a framework for understanding the psychology of threat appeals. It offers an explanation as to why certain studies find threat appeals to be effective in reducing risky behavior, some find no impact, and others report ‘boomerang’ results, where threat appeals lead to an increase in maladaptive behavior. The EPPM proposes that it is threat-by-efficacy interactions that determine the outcome of threat appeal studies. Specifically, the author posits that the impact of threat appeals on behavioral outcomes is determined by perceived severity of the threat, perceived susceptibility to the threat, and perceived efficacy.

Perceived efficacy, Witte proposes, is made up of response efficacy (i.e. beliefs about the effectiveness of the recommended behavior, for example, ‘driving slowly is an effective way to avoid road traffic collisions’) and self-efficacy (i.e. beliefs about one's ability to carry out the recommended behavior, for example, ‘I could drive slowly and in that way reduce my chances of being involved in a road traffic collision’). According to the EPPM, when a threat is sufficiently severe (e.g. the consequences of the depicted road traffic collision involve serious injury), and perceived efficacy is high (e.g. people are advised to drive more slowly, and they believe this strategy to be effective and achievable by them), people will be motivated to control the threat by engaging in adaptive, protective actions (danger control). In other words, they will adopt the recommended response (i.e. drive more slowly). On the other hand, when the threat is too high, and/or the perceived efficacy too low, people will respond by engaging in maladaptive psychological defense mechanisms (fear control). They may not change their behavior, or they may change it in a maladaptive way.

One important point about the EPPM, particularly in the context of the current paper, is the emphasis it places on the role of fear. While earlier models assumed fear to be an indirect or insignificant construct in threat appeal theory, the EPPM assigns it a more prominent role [11]. As discussed later in this paper, the role of fear is one that has been largely understated in the empirical threat appeal literature. By incorporating both fear control and danger control processes, the EPPM provides a comprehensive account of psychological responses to threat appeals. Since its development, the EPPM has been adopted in a number of areas related to health promotion, and is the most widely accepted threat appeal-specific framework in the literature currently [26]. Despite proposals to refine the model by adding additional variables, and accounting for cultural differences, the model remains largely unchanged since its development [27].

While perceived efficacy and perceived severity/susceptibility are the key variables to emerge from the EPPM, other theoretical models have highlighted the role of additional factors that may influence the impact of threat appeals on behavior. For example, Terror Management Theory [TMT; 28] has been applied to threat appeal research in a number of recent papers [3], [4], [10], [29], and emphasizes self-esteem as an important factor in moderating the effectiveness of threat appeals. Specifically, TMT suggests that, among individuals who view driving as a source of self-esteem, death-related threat appeals may provoke defensive responses, in certain cases leading to an increase in the risky driving behavior (for a recent meta-analysis of TMT research, see [30]).

### The current study

Key points to emerge from these models, and from existent empirical research in general, is that the underlying psychological mechanisms at play in the fear-behavior relationship are likely to be complex, and do not always involve direct causation. Rather, there are likely to be moderators and mediators of this relationship, and a key task for researchers and advertisers is to identify and better understand these factors.

Despite the numerous relevant reviews and meta-analyses, there has been no attempt to systematically identify and synthesize experimental, cause-effect studies in the threat-based road-safety literature, the findings of which would offer a source of more conclusive evidence. The current paper presents the results of the first meta-analysis of the experimental research on threat appeals and driving. Specifically, we use meta-analytic techniques to examine the impact of threat-based messages on fear arousal and lab-based indices of driving behavior.

Threat appeals are incorporated into health promotion campaigns under the assumption that they elicit anticipatory fear of experiencing a negative outcome in the audience, and that the audience subsequently responds by adopting healthy behaviors and/or avoiding risky ones [31]. That is, threat elicits fear, and fear results directly in behavioral avoidance or modification. However, despite detailed theoretical models and numerous experimental studies and reviews, the role of fear in the threat appeal literature remains unclear [32]. Since a certain level of fear arousal is seen as a prerequisite for threat appeals to work, one aim of the current paper is to examine if and how the included studies measured fear, whether or not the study manipulations had a significant impact on fear, and if emotions other than fear, such as disgust, were controlled for in the included studies.

A second aim of the analysis is to examine the causal impact of threat appeals on driving behavior. Drawing on a recent meta-analysis by Gerber and Wheeler [33], we included findings only from experimental paradigms, which, in cases like these can be “more meaningful and interpretable” (p. 472). In order to compare the different types of outcome variables used across studies in this area, the current analysis examines the impact of threat appeals on three distinct indices of driving: self-reported driving intentions, simulated driving and scores from a VST.

The threat-fear-behavior relationship is complex, and a number of influential factors have been identified by previous research, including perceived severity/susceptibility and perceptions of efficacy. A third aim of the current meta-analysis is to identify the known moderators and mediators of the relationship between fear and driving outcomes.

## Methods

### Inclusion criteria and selection procedures

The broader threat appeal literature from the 1960s–1990s has been widely reviewed, with numerous papers providing conceptual and methodological analyses of this body of research [2], [19], [34], [35], [36], [37]. With the aim of advancing meta-analytic research in this area, the current analysis covers the period from 1990 to 2011. A second reason for restricting the review to this time period is that mass media campaigns have evolved over time, and this meta-analysis sought to include studies that used exposure material likely to resonate with current threat-appeal message design.

In order to establish an estimate of the causal impact of threat appeals on behavior, and in line with several recent meta-analyses [38], [39], experimental control was an important factor in our inclusion criteria. All included studies therefore adopted an experimental design. We conducted a search of previous reviews and meta-analyses of the general threat appeal literature. A comprehensive search of electronic databases was also completed, including EBSCO, PubMed, PsycArticles, PsycInfo, Sage, MEDLINE and ScienceDirect. Keywords included ‘threat appeals’, ‘fear appeals’, ‘scare tactics’, ‘advertisements’, ‘road safety’ and ‘risky driving’ [e.g. Driv* AND (fear appeals OR threat appeals OR road safety campaigns)]. We trawled key journals in the area (e.g. *Accident Analysis and Prevention*, *Transportation Research Part F* and the *Journal of Personality and Social Psychology*), as well as the bibliographies of relevant articles. In order to address the so-called file-drawer problem [40], where unpublished studies are considered to represent the 95% of studies that show non-significant results, we searched the grey (unpublished) literature through the ProQuest database. We accessed conference abstracts and also contacted leading authors in the field to request relevant unpublished material.

### Data extraction

**Figure 1:**
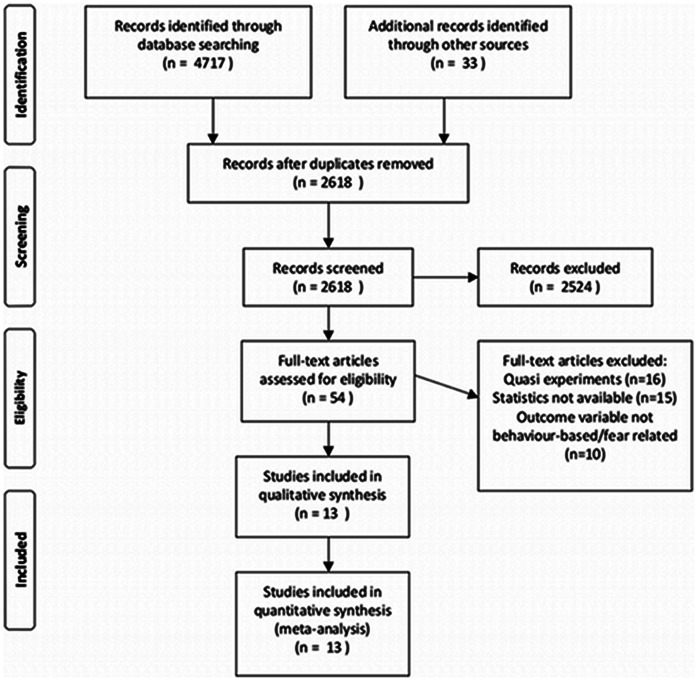
PRISMA Flow Diagram outlining identification and selection procedures.

Figure 1 presents a graphical representation of the selection procedures. An initial review of the titles and abstracts of papers led to the identification of 54 articles that met our inclusion criteria. The full-text articles were accessed and reviewed. 16 lacked a control group and/or control of potential confounds, and were excluded. The remaining 38 were ‘true’ experiments (clear, controlled manipulation of potential confounds and inclusion of a control group). Fifteen of these were subsequently eliminated, as they did not provide the statistics (nor could the statistics be accessed from the authors) necessary to compute effect sizes. Of the remaining 23 studies, 13 examined the difference in behavior-based dependent variables between participants exposed to threat appeals, and those in a control group. Results presented here are based on the analysis of these 13 studies (see Table 1).

**Table 1:** Characteristics of included studies.

First-named author	Included *N*	P/UP	Age	Location	MO/mixed	Type of risky driving	Outcome variable measured
**Carey ** [**3**]	80	P	17–24	IRE	MO	Various	SRI
**Chen ** [**68**]	200	UP	18–30	USA	Mixed	Talking on cell	FA, SRI
**Goldenbeld ** [**69**]	81	P	M = 51	NL	Mixed	Speed	SRI
**Jessop ** [**4**]	199	P	18–30	UK	Mixed	Various	SRI
**Lennon ** [**70**]	673	P	M = 21.6	USA	Mixed	Various	SRI
**Nielsen ** [**71**]	168	P	17–24	USA	Mixed	Drink Driving	FA
**Rosenbloom ** [**72**]	120	P	20–33	Israel	Mixed	Various	SRI
**Shehryar ** [**73**]	178	UP	M = 22.8	USA	Mixed	Drink Driving	FA
**Taubman** **Ben-Ari ** [**67**]	603	P	18–21	Israel	MO	Various (S1 & 2)Speed (S3 & 4)	SRI (S1 & 2)DSS (S3 & 4)
**Taubman** **Ben-Ari ** [**12**]	109	P	18–21	Israel	MO	Various (S1), Speed (S2)	SRI (S1), DSS (S2)
**Taubman** **Ben-Ari ** [**10**]	206	P	18–21	Israel	Mixed	Various	SRI
**Thornton ** [**74**]	354	UP	17–28	AUS	Mixed	Speed	VST
**Yaoshan ** [**75**]	73	UP	NS	China	MO	Various	FA, SRI

*Note: P = Published, UP = Unpublished, MO = Males Only, SRI = Self-reported intentions, DSS = driving simulator speed, VST = video speed test, FA = fear arousal.*

### Coding and analysis

All studies were independently coded by two raters. Agreement on coding was 95% and differences were resolved through discussion. Studies were coded according to outcome variables, type of risky driving (where applicable), modality of exposure (i.e. how the manipulation was presented to participants; video-based or still image/fact-based), as well as gender, age (ages 17–24, 25–30 and 30 plus), whether or not the study was published, year of the study, country of origin, theoretical framework, inclusion of a fear arousal measure, whether or not a follow-up test was carried out and whether or not previous risky driving behavior/crash history had been measured.

Statistical values (i.e., *p*, *t*, means and standard deviations) were extracted from the identified studies and converted to correlation effect sizes (i.e., *r*). Calculation of the weighted average effect size, as well as all other computations, was performed using the software *Comprehensive Meta-Analysis* (CMA; Copyright ©2006 Biostat, Inc.). For all analyses, the threat appeal group was compared with the control group. Where there were multiple levels or trials of an outcome variable in a study, one average effect size per outcome variable per study was calculated. Effects were not averaged in a study when they related to different outcomes.

According to Fern and Monroe [41], effect sizes can “only be unequivocally interpreted when the research uses a random-effects model” (p. 95). Due to wide range of studies, and the importance of generalizing our findings beyond the included studies, random effects models were chosen as the computational model across all analyses. In contrast to the fixed effects model, the random effects model does not assume that all of the studies in the meta-analysis are functionally equivalent, allowing the true effect sizes to differ [42], [43].

The 13 studies included in the meta-analysis contributed 71 effect sizes and a total sample size of 3044 (1894 males, 972 females, 178 unreported). A majority of the studies (*k* = 9) used both male and female participants, while 4 used a male-only sample.

### Assessing heterogeneity and publication bias

Heterogeneity analyses were conducted using Cochran's [44] *Q-*statistic, as well as Higgins and Thompson's [45] *I* ^2^ index. A significant *Q*-statistic suggests that the hypothesis of homogeneity should be rejected, while the *I* ^2^ index represents a value of heterogeneity in percentage form. According to Higgins and Thompson, 25% suggests low heterogeneity, 50% suggests medium heterogeneity and 75% or higher represents high heterogeneity. Tests of heterogeneity were significant, as expected, with the *I^2^* value indicating high heterogeneity in the studies (*p*<.001, *I^2^*>75%). This result further justifies the adoption of the random effects model in the current analyses.

Funnel plot analyses [46] and Duval and Tweedie's trim and fill procedure [47], carried out by the *CMA* software, revealed no evidence of publication bias (*r* = .04, [95% CI: .01–.09], number of studies trimmed = 0).

## Results

### Analysis of threat appeals fear arousal and driver behavior

Theoretically, threat appeals aim to modify driving by increasing fear of negative outcomes. Thus, we first looked for evidence that threat exposures were resulting in an elevated fear response. Four of the included studies measured fear as an outcome (all used a form of self-report scale). When the 4 studies that included fear arousal as a dependent variable were analyzed, large effects emerged, with experimental groups reporting increased fear arousal in comparison to control groups (*r* = .64, *n* = 619, *p*<.01). None of the included studies controlled for emotions other than fear which may have been evoked by the message, the importance of which is highlighted by previous research [48], [49].

Subsequently, we investigated the overall effect of threat appeals on driving behavior, not differentiating between simulated, self-report or VST outcome variables. No significant effect of threat appeals on the driving outcome variables emerged (*r* = .03, *n* = 2425, *p* = .17 [95% CI: −.01 to .07]). When we examined each outcome variable separately, we found no significant difference between threat appeal/control groups on self-reported intention to take driving risks (*r* = .02, *n* = 2125, *p* = .38 [95% CI: −.03 to .07]) or in driving simulator speed or speed during a VST (*r* = .08, *n* = 573, *p* = .26 [95% CI: −.06 to .21]).

Studies using a video-based manipulation produced particularly strong effects on fear (*r* = .64, *n* = 273, *p*<.05), with no effect on driving behavior/intentions (*r* = −.01, *n* = 1610, *p* = .62). The studies included used male-only or mixed samples. Focusing on males, (*k* = 5, *n* = 925) the threat-appeals had no impact on this sub-sample (*r* = .05, *p* = .06). Since there were no studies that used a female-only sample, we cannot compare across genders.

### Moderator analyses

No moderator of the impact between threat and driving outcomes emerged consistently across a majority of studies included in this meta-analysis. Consequently, it was not possible to conduct a moderator analysis.

Of the 13 studies, 11 included moderators or mediator variables in their analysis, reflecting four distinct theoretical positions (Terror Management Theory, [28], Protection Motivation Theory, [50], the fear-as-acquired drive (drive-reduction) model, [51] and the EPPM, [24]). The variables were self-esteem, perceived severity, perceived susceptibility, perceived response efficacy and self-efficacy, ego involvement, perceived behavioral control, social norms, the third-person effect, fear pattern, group discussions and the personality trait of sensation seeking. Significant moderators within studies included self-esteem [3], [4], [10], [12], perceived severity, susceptibility, perceived response efficacy and perceived self-efficacy [29], and these findings are considered further later in this paper.

## Discussion

The findings of this meta-analysis suggest that, while threat appeals can have a strong impact on the level of fear aroused in individuals, they do not reliably impact on behavior. This finding points to the complexity of the relationship between emotion and behavior, a relationship that is poorly understood in the threat appeal literature. The link between threat appeals, elicited fear and behavior is widely debated, yet unresolved.

There is a lack of consistency in how fear is defined, treated and interpreted in threat appeal studies. Only 4 of the 13 studies in the current analysis measured fear as an outcome. As pointed out by Lewis, Watson and White [52], a measure of fear arousal can provide important information about the emotions evoked by the appeal, as well as serving as a manipulation check. The assumption that a threat message evokes fear is a common one. Our findings suggest that the impact of threat appeals on fear is strong, but it is important that this effect is measured and analyzed, rather than assumed, in individual experimental studies.

Of the studies that measured fear as an outcome variable, all used some form of self-report scale (e.g. Likert Scales, the Positive and Negative Affect Scale [53]). This reflects points raised by other researchers who suggest that, where fear has been given a central role in experimental studies, the ways of measuring it have been inconsistent [54]. Problems associated with fear arousal measures in threat appeal studies have been highlighted by researchers for decades. For example, in one of the earliest influential reviews of the literature [54], Higbee points out that there are large differences in the way fear has been measured in the literature, from self-reported anxiety measures, to reported worry or concern. More recently, Matsumoto and colleagues [55] point out that, since emotions are transitory, and thus can change substantially in a number of seconds, self-report measures may not be capable of capturing the complexity of the emotional experience.

One way of addressing the limitations posed by self-report measures of fear is by measuring fear *objectively*, using physiological measures such as heart rate monitoring and skin conductance responses [32], or a continuous measurement dial [56]. We suggest that threat appeal messages should be systematically and comprehensively piloted to examine their impact on objective measurements of fear, before being used in experimental studies.

A further limitation of the included studies is that emotions, other than fear, evoked by the message, were not controlled for. Threat messages can elicit emotions such as guilt, shame and anger, and researchers have recognized that the interplay between the different emotions can determine the effectiveness of the message [57], [58]. Specifically, several studies have addressed the need for a distinction between the related emotions of fear and disgust, when examining responses to threat-based stimuli [48], [59]. Again, this can be controlled for by a comprehensive piloting procedure, prior to the experimental study.

No significant difference was found between studies that used self-reported driving intentions as the outcome variable, and those that used driving simulators or video-based speed tests. Recent studies have begun to test the validity of in-vehicle data recording devices in cars, which provide a measure of realistic driving behavior [60]. These in-vehicle devices have promising potential in the measurement of driving behavior, and are likely to become the gold standard in future studies in the area.

Experiments using male-only samples showed particularly weak effects for threat appeals on driving behavior. As emphasized by Lewis et al. [11], such findings represent a major challenge to road safety practitioners and researchers, particularly since young male drivers tend to engage in more risky and less cautious driving than females [e.g. 61]. The main target audience of many road safety campaigns may be the audience least influenced by them. The next step for research should be to determine the profile of individuals who are resilient to threat appeal messages, and to determine the types of messages that are likely to bring about a change in behavior among this population.

Overall, our findings present further evidence that the link between fear and behavior is complex, and likely to be moderated or mediated by other factors. Previous meta-analyses in the area of threat appeals or risky driving have included moderator variables such as trait anxiety [2], the context of the accident [62] and the type of driver improvement intervention [63]. A recent meta-analysis reported that threat and efficacy interact in their effects on behavior. The authors noted that threat appeals had an effect only if there was high efficacy in the message, and efficacy had an effect only when the message was high in threat [5]. In a test of the EPPM, Witte [64] found that, overall, the fear emotion lead to threat appeal failure (through fear control processes), while cognitions lead to their success (through danger control processes).

Recent research from the broader threat appeal literature has highlighted the importance of a number of such moderators and mediators, including the perceived severity of the threat, the individual's perceived vulnerability to the threat, the perceived efficacy of the recommended response, as well as the individual's beliefs surrounding their ability to carry out that response [65], [66]. These variables were measured in one of the included studies [29] and findings suggested that, while they are useful in explaining responses to non death-related threats, TMT variables such as ego-involvement are of more use in explaining responses to death-related threats. These findings suggest that including TMT variables, such as ego-involvement and self-esteem, may help add to the explanatory power of threat appeal studies. Five of the studies included in the current analysis found self-esteem to significantly moderate the impact of threat appeals on behavior, such that individuals who derived self-esteem from driving reported higher risky driving intentions following a mortality salient (death-related) prime [3], [4], [10], [12], [67]. Again, this suggests that including variables such as self-esteem in experimental designs may help improve our understanding of psychological responses to threat appeals.

From reviewing the included studies, as well as the broader literature base, it seems likely that cognitions are moderating the impact of threat appeals on behavior outcomes. Incorporating EPPM variables (i.e. perceived efficacy and perceived severity/susceptibility), and TMT variables (e.g. self-esteem) into threat appeal research may help advance current knowledge and improve predictive power. The recent work by Baumeister and colleagues [21], [22] also offers an insight into the emotion-cognition-behavior relationship, and this may prove useful in informing future research.

## Conclusions

The current meta-analysis aimed to examine the impact of threat appeals on fear arousal and on behavior-based/self-reported driving outcomes. Our findings suggest a disconnect between emotion (i.e. fear) and behavior (i.e. driving) - a disconnect that is reflected in the inconsistent findings in the threat appeal driving literature. We suggest that these inconsistent research findings are likely to reflect a lack of sophistication surrounding our understanding of the link between emotion and action. It is possible that experimental research has not, as of yet, adopted the types of complex conceptualizations of the fear-behavior relationship that are necessary to yield valid, replicable findings.

While strong evidence exists to suggest that threat-based messages are effective under the right conditions, based on findings to date, there is little evidence to suggest that they consistently work. Males continue to represent the at-risk group for RTCs, and are also those most resilient to threat appeals.

What is now needed, in order for the contributions of experimental science to this area to be applied to policy and campaign design, is for researchers to a) adopt complex theoretical positions and b) adopt designs that can test these theories in full. This means that emotional responses, both fear and other emotions, need to be measured or statistically controlled for, preferably through a comprehensive piloting procedure.
